# The Serum NLRP1 Level and Coronary Artery Calcification: From Association to Development of a Risk-Prediction Nomogram

**DOI:** 10.31083/j.rcm2507265

**Published:** 2024-07-16

**Authors:** Jingfeng Peng, Bihan Zhou, Tao Xu, Xiabing Hu, Yinghua Zhu, Yixiao Wang, Siyu Pan, Wenhua Li, Wenhao Qian, Jing Zong, Fangfang Li

**Affiliations:** ^1^Department of Cardiology, The Affiliated Hospital of Xuzhou Medical University, 221000 Xuzhou, Jiangsu, China; ^2^Institute of Cardiovascular Disease Research, Xuzhou Medical University, 221000 Xuzhou, Jiangsu, China; ^3^Department of Electrocardiography, The Affiliated Tumor Hospital of Nantong University, Nantong Tumor Hospital, 226000 Nantong, Jiangsu, China

**Keywords:** coronary artery calcification, coronary artery disease, NLRP1, prediction model, nomogram

## Abstract

**Background:**

To investigate the correlation between inflammasomes and coronary artery calcification (CAC), and develop and validating a nomogram for predicting the risk of CAC in patients with coronary artery disease (CAD).

**Methods:**

A total of 626 patients with CAD at the Affiliated Hospital of Xuzhou Medical University were enrolled in this study. The patients were divided into the calcification group and the non-calcification group based on the assessment of coronary calcification. We constructed a training set and a validation set through random assignment. The least absolute shrinkage and selection operator (LASSO) regression and multivariate analysis were performed to identify independent risk factors of CAC in patients with CAD. Based on these independent predictors, we developed a web-based dynamic nomogram prediction model. The area under the receiver operating characteristic curve (AUC-ROC), calibration curves, and decision curve analysis (DCA) were used to evaluate this nomogram.

**Results:**

Age, smoking, diabetes mellitus (DM), hyperlipidemia, the serum level of nucleotide-binding oligomerization domain (NOD)-like receptor protein 1 (NLRP1), alkaline phosphatase (ALP) and triglycerides (TG) were identified as independent risk factors of CAC. The AUC-ROC of the nomogram is 0.881 (95% confidence interval (CI): 0.850–0.912) in the training set and 0.825 (95% CI: 0.760–0.876) in the validation set, implying high discriminative ability. Satisfactory performance of this model was confirmed using calibration curves and DCA.

**Conclusions:**

The serum NLRP1 level is an independent predictor of CAC. We established a web-based dynamic nomogram, providing a more accurate estimation and comprehensive perspective for predicting the risk of CAC in patients with CAD.

## 1. Introduction

Coronary artery disease (CAD) is a cardiovascular disease (CVD), manifested by stable angina, unstable angina, myocardial infarction, or sudden cardiac death, and is one of the primary causes of death worldwide [1]. Despite advances in diagnostic and treatment technologies in recent years, the prevalence of CAD continues to increase annually, and represents a serious threat to public health [2]. Vascular calcification, especially coronary artery calcification (CAC), is prevalent, harmful, and progresses rapidly in patients with CAD. Previous studies have shown that the presence of CAC increases the risk of coronary heart disease events by threefold [3]. Pathological studies have demonstrated a strong correlation in the initiation and progression between CAC and CAD [4]. CAC is often located in areas of atherosclerotic lesions [5]. The severity of CAC and the degree of coronary stenosis directly impact the management and treatment of CAD. In addition, it is difficult to perform treatments to eradicate CAC. Thus, early anticipation of the high risk of CAC and timely intervention are pivotal for the treatment and prognosis of patients with CAD.

The conventional risk factors, such as race, advanced age, male gender, smoking, diabetes mellitus (DM), hypertension, hyperlipidemia, and chronic kidney disease (CKD), associated with the presence and development of CAC have been widely recognized in the general population [3, 6, 7, 8]. In previous views, the formation of CAC was believed to be caused by the ectopic deposition of calcium salts in the walls of coronary vessels, which was considered as a passive and degenerative pathological phenomenon. However, recent studies support a concept that CAC is an active and regulated process in atherosclerosis progression, reflecting a broader systemic inflammatory response [5, 6]. A study demonstrated that as atherosclerosis progresses, inflammation aids in the initiation and progression of calcification as macrophages secrete inflammatory cytokines and promote osteogenic differentiation of vascular cells [9]. Inflammasomes derived from macrophages, can be activated by various cardiovascular risk factors and drive downstream signaling events. Studies have shown that the inflammasomes nucleotide-binding oligomerization domain (NOD)-like receptor protein 1 (NLRP1) and NLRP3 are closely associated with CVD [10, 11]. Therefore, the inflammasomes NLRP1 and NLRP3 may be able to potentially detect high-risk populations and improving the ability to predict the occurrence of CAC.

The aim of this study was to determine whether the inflammasomes NLRP1 and NLRP3 could serve as a “risk integrator” for CAC, adding predictive information beyond conventional cardiovascular risk factors. Since there is a paucity of research in this area, we conducted a clinical data analysis to assess the relationship between NLRP1, NLRP3 and CAC. We developed a simple and cost-effective nomogram-illustrated model aiming to predict the occurrence of CAC in patients with CAD and to improve therapeutic decisions leading to the primary prevention of CAC.

## 2. Methods

### 2.1 Study Population

The study cohort comprised 744 patients with suspected CAD who underwent coronary angiography (CAG) in the Cardiology Department of Xuzhou Medical University Affiliated Hospital from January 2021 to March 2023. The research was approved by the Ethics Committee of the Affiliated Hospital of Xuzhou Medical University (No: XYFYLW2017-002), and all participants provided written informed consent.

**Fig. 1.**
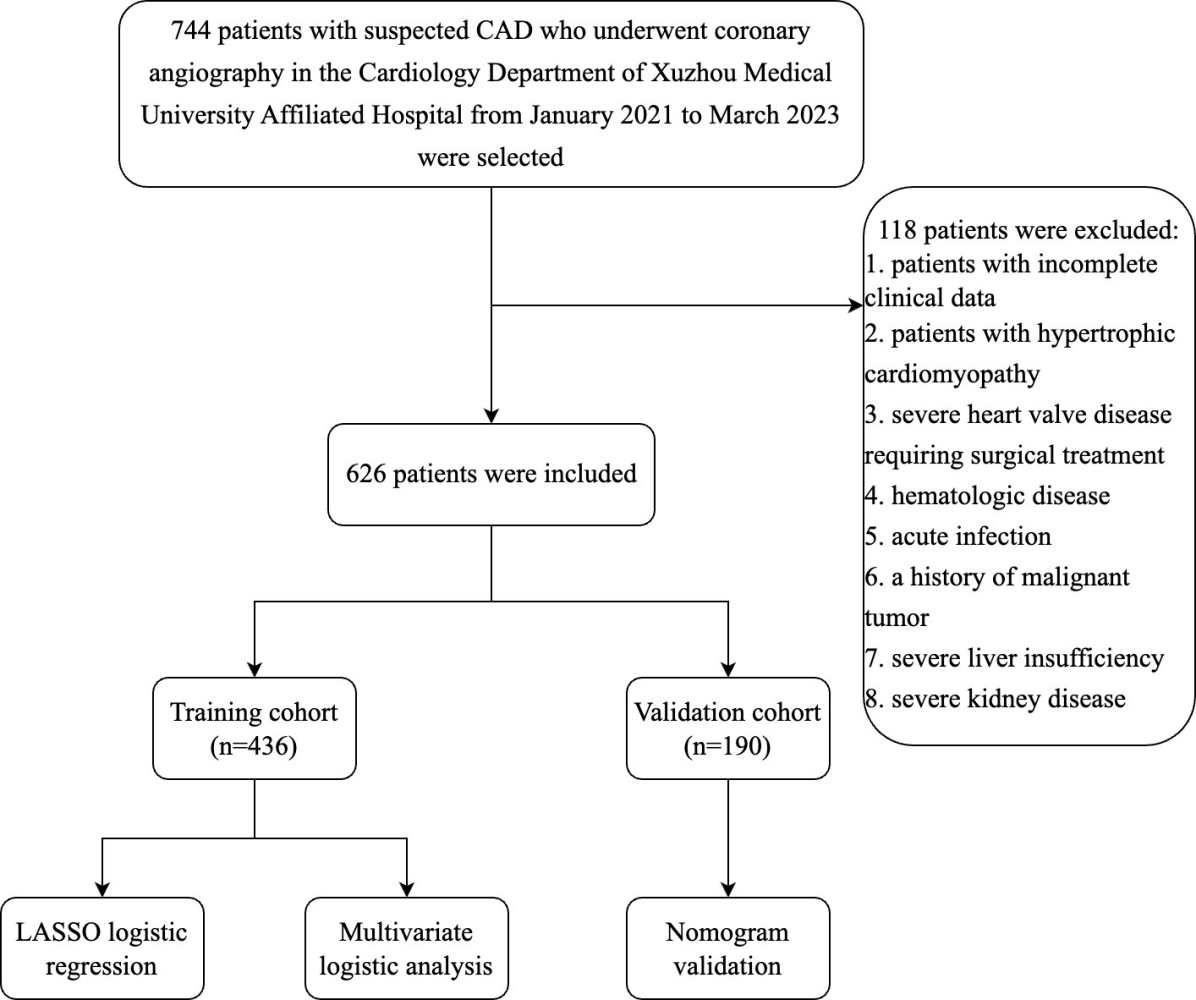
**Flow chart of the inclusion and exclusion process of all patients enrolled in this study.** Abbreviation: CAD, coronary artery disease; LASSO, least absolute shrinkage and selection operator.

The criteria for inclusion were: patients with myocardial ischemic symptoms, clinically suspected diagnosis of CAD, agreement to undergo CAG. Based on the results of CAG, CAD was diagnosed as coronary stenosis (≥50%) in at least one major coronary vessel. The exclusion criteria were: (i) patients with incomplete clinical data, (ii) patients suffering from hypertrophic cardiomyopathy, (iii) severe heart valve disease requiring surgical treatment, (iv) hematologic disease, (v) acute infection, (vi) malignant tumor, (vii) severe liver insufficiency, and (viii) severe kidney disease (estimated glomerular filtration rate (eGFR) <15 mL/min/1.73 $m^{2}$). The flow chart of the inclusion and exclusion process is shown in Fig. 1.

### 2.2 Clinical Data Collection

Patient clinical data were collected by reviewing electronic medical records, including demographic data (age, gender, body mass index (BMI)), past medical history (hypertension, DM, hyperlipidemia), laboratory indicators (white blood cell (WBC), neutrophil (NE), lymphocyte (LY), platelet (PLT), high-sensitivity C-reactive protein (hsCRP), aspartate aminotransferase (AST), alanine transaminase (ALT), alkaline phosphatase (ALP)) and medications (statin, Ezetimibe, aspirin, clopidogrel angiotensin-converting enzyme inhibitor (ACEI), oral hypoglycemic drugs, insulin). All biochemical tests were completed 24 hours after admission.

### 2.3 NLRP1 and NLRP3 Measurements

The serum levels of NLRP1 and NLRP3 were detected with ELISA kits purchased from CUSABIO BIOTECH Co., Ltd (Wuhan, Hubei, China). All blood samples were collected and tested within 24 hours after admission.

### 2.4 Coronary Calcium Detection

The presence of CAC was assessed during CAG procedures by accredited interventional cardiologists. Since the accuracy of CAG to identify CAC is suboptimal, the majority of individuals also underwent intravascular ultrasound (IVUS) during the procedure to identify CAC [6].

### 2.5 Statistical Analysis

We used SPSS (version 22.0, IBM Corp., Armonk, NY, USA) and R software (version 3.6.4, R Foundation for Statistical Computing, Vienna, Austria) for statistical analysis. Categorical variables were expressed as counts and percentages (%) and compared using the χ^2^ test. The normality and homogeneity of continuous variables were assessed using the Shapiro-Wilk test and the Levene’s test. The data conforming to a normal distribution was represented as mean ± standard deviation ($\bar{x}$ ± s), and comparison between groups was conducted using an independent sample *t*-test. Continuous variables without a normal distribution were presented as medians (*M*) and interquartile ranges *M *(P25, P75), and were compared by accessing the nonparametric test. A *p* < 0.05 was considered statistically significant. Multiple binary logistic regression analysis, and the Backward Wald method, was used to define the independent predictors of CAC. Additionally, based on the 10 Events Per Variable rule, the sample size in this clinical study was sufficient, and therefore a sample size calculation was not performed. The least absolute shrinkage and selection operator (LASSO) regression was used for screening potential predictors of CAC by reducing the dimensions of the characteristics that were selected. Then, the selected variables were incorporated into a multivariate regression analysis to determine whether they were independent predictors of CAC. The nomogram was established by introducing these independent predictors into R software. We performed an internal validation using the Bootstrap method. Finally, the discriminative ability, calibration and clinical value of the nomogram were evaluated respectively by the area under the receiver operating characteristic curve (AUC-ROC), calibration curves, and decision curve analysis (DCA).

## 3. Results

### 3.1 Baseline Characteristics

Among the total of 626 patients included in this study, 338 had coronary calcification. The differences in characteristics between the non-calcification group and the calcification group are shown in Table 1. Compared with the non-calcification group, patients in the calcification group tended to be older, obese, smokers and were more likely to suffer from hypertension, DM, and hyperlipidemia. The calcification group also had higher NLRP1, hsCRP, ALP, fasting blood glucose (FBG), triglyceride (TG), small dense LDL-cholesterol (sdLDL), N-terminal-pro brain natriuretic peptide (NT-proBNP), glycated hemoglobin A (HbA1c) and lower ALT, apolipoprotein A (APOA1), and eGFR levels. Ticagrelor and beta-blockers were used more frequently in the calcification group.

**Table 1.** **Baseline characteristics of the non-calcification group and calcification group**.

Variables		Non-calcification group	Calcification group	*p-*Value
		(n = 288)	(n = 338)	
Age		54.60 ± 9.35	69.81 ± 8.13	<0.001
Gender (n, %)				0.074
	Male 1	173 (60.1%)	179 (53.0%)	
	Female 2	115 (39.9%)	159 (47.0%)	
BMI, ${\mathrm{kg/m}}^{2}$		25.71 (23.33, 28.03)	24.97 (22.60, 27.13)	0.007
Smoking (n, %)				0.027
	No	208 (72.2%)	216 (63.9%)	
	Yes	80 (27.8%)	122 (36.1%)	
Drinking (n, %)				0.115
	No	202 (70.1%)	256 (75.7%)	
	Yes	86 (29.9%)	82 (24.3%)	
Past medical history (n, %)				
	Hypertension	142 (49.3%)	200 (59.2%)	0.013
	DM	72 (25%)	137 (40.5%)	<0.001
	Hyperlipidemia	80 (27.8%)	126 (37.3%)	0.012
Hematological indicators				
	NLRP1, pg/mL	42.61 (25.57, 61.54)	55.86 (32.35, 74.67)	<0.001
	NLRP3, pg/mL	42.31 (20.20, 71.99)	41.78 (19.59, 70.82)	0.993
	WBC, ${\mathrm{10}}^{9}$/L	5.90 (4.90, 7.10)	5.70 (4.80, 7.20)	0.898
	NE, ${\mathrm{10}}^{9}$/L	3.51 (2.78, 4.53)	3.52 (2.80, 4.46)	0.960
	LY, ${\mathrm{10}}^{9}$/L	1.70 (1.30, 2.10)	1.60 (1.30, 2.00)	0.158
	PLT, ${\mathrm{10}}^{9}$/L	212.00 (184.50, 251.75)	212.00 (169.00, 244.00)	0.117
	hsCRP, mg/L	1.60 (0.80, 2.70)	1.65 (0.50, 6.50)	0.038
	AST, U/L	18.00 (16.00, 24.75)	18.00 (15.00, 24.00)	0.549
	ALT, U/L	18.00 (13.00, 28.75)	16.00 (11.00, 25.00)	0.005
	ALP, U/L	68.00 (58.00, 85.00)	77.00 (63.00, 99.25)	<0.001
	Urea, mmol/L	5.16 (4.25, 6.12)	5.10 (4.28, 6.34)	0.393
	Scr, umo/L	62.00 (53.00, 70.00)	62.00 (54.00, 74.00)	0.144
	UA, umo/L	301.00 (255.25, 368.00)	299.00 (239.00, 365.25)	0.403
	eGFR, mL/min	107.82 (95.91, 119.18)	102.61 (88.16, 116.13)	<0.001
	FBG, mmol/L	5.61 (5.15, 6.40)	5.80 (5.25, 7.41)	0.003
	TC, mmol/L	4.11 (3.24, 5.11)	4.09 (3.48, 4.90)	0.794
	TG, mmol/L	1.47 (1.02, 2.04)	1.68 (1.34, 2.22)	<0.001
	LDL-C, mmol/L	2.51 (1.84, 3.08)	2.62 (1.75, 3.29)	0.553
	HDL-C, mmol/L	1.10 (0.94, 1.28)	1.06 (0.92, 1.24)	0.146
	APOA1, g/L	1.28 (1.11, 1.52)	1.21 (1.07, 1.44)	0.017
	APOB, g/L	0.87 (0.77, 1.04)	0.93 (0.76, 1.11)	0.309
	Lp (a), mg/L	216.00 (150.25, 339.50)	259.50 (163.75, 358.75)	0.267
	sdLDL, mmol/L	0.59 (0.43, 0.90)	0.71 (0.48, 1.01)	0.005
	NT-proBNP, pg/mL	121.41 (99.00, 284.51)	192.61 (99.00, 725.03)	<0.001
	HbA1c, %	5.90 (5.60, 6.68)	6.10 (5.70, 6.73)	0.003
	LVEF, %	60.00 (55.61, 62.99)	59.48 (56.24, 63.09)	0.510
Inotropic drugs (n, %)				
	Statin	266 (92.4%)	312 (92.3%)	0.980
	Ezetimibe	51 (17.7%)	62 (18.3%)	0.837
	Apolizumab	41 (14.2%)	51 (15.1%)	0.764
	Aspirin	279 (96.9%)	328 (97.0%)	0.904
	Clopidogrel	86 (29.9%)	113 (33.4%)	0.339
	Ticagrelor	157 (54.5%)	217 (64.2%)	0.014
	ACEI/ARB	81 (28.1%)	107 (31.7%)	0.337
	ARNI	53 (18.4%)	66 (19.5%)	0.721
	Beta-blockers	157 (54.5%)	217 (64.2%)	0.014
	CCB	51 (17.7%)	60 (17.8%)	0.989
	Oral hypoglycemic drugs	85 (29.5%)	108 (32.0%)	0.510
	Insulin	17 (5.9%)	21 (6.2%)	0.871

Abbreviations: BMI, body mass index; DM, diabetes mellitus; NLRP1, nucleotide-binding oligomerization domain like receptor protein 1; NLRP3, nucleotide-binding oligomerization domain like receptor protein 3; WBC, white blood cell; NE, neutrophil; LY, lymphocyte; PLT, platelet; hsCRP, high-sensitivity C-reactive protein; AST, aspartate aminotransferase; ALT, alanine transaminase; ALP, alkaline phosphatase; Scr, serum creatinine; UA, uric acid; eGFR, estimated glomerular filtration rate; FBG, fast blood glucose; TC, total cholesterol; TG, triglyceride; LDL-C, low-density lipoprotein cholesterol; HDL-C, high-density lipoprotein cholesterol; APOA1, apolipoprotein A; APOB, apolipoprotein B; Lp (a), lipoprotein (a); sdLDL, small dense low density lipoprotein; NT-proBNP, N-terminal-pro brain natriuretic peptide; HbA1c, glycated hemoglobin A; LVEF, left ventricular ejection fraction; ACEI, angiotensin-converting enzyme inhibitor; ARB, angiotensin receptor blockers; ARNI, angiotensin receptor neprilysin inhibitor; CCB, calcium channel blocker; n, the number of patients per group.

### 3.2 LASSO Regression and Multivariate Logistic Regression Analysis

**Fig. 2.**
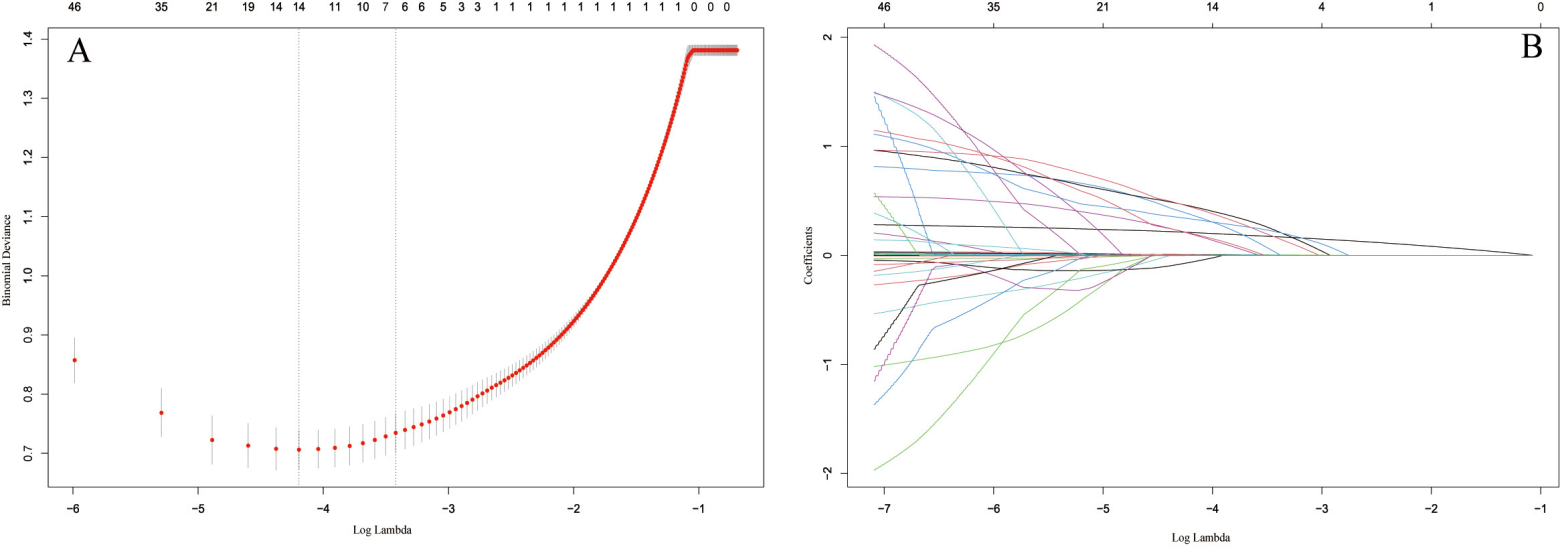
**Identification of the predictors by LASSO regression.** (A) The cross-validation plot. 7 variables were identified by selecting optimal value (λ = 0.03266). (B) LASSO regression coefficient plot. 7 variables that remained in the model the longest as the penalization increased. Abbreviation: LASSO, least absolute shrinkage and selection operator.

As shown in Fig. 2, by selecting the optimal lambda value, the LASSO regression assisted us in determining 7 candidate variables from an initial set of 46. After adding the variables into the multivariate logistic analysis, the results indicated that age, BMI, smoking, DM, hyperlipidemia, NLRP1 and ALP were independent risk factors for CAC in patients with CAD (Table 2).

**Table 2.** **Multivariate logistic analysis for the CAC**.

Variables		β	s$\bar{\chi }$	Wald χ^2^	OR (95% CI)	*p-*Value
Age		0.267	0.026	102.097	1.307 (1.241, 1.376)	<0.001
Smoking		0.905	0.349	6.716	2.471 (1.247, 4.898)	0.01
Past medical history						
	Diabetes	0.84	0.332	6.413	2.172 (1.062, 4.442)	0.011
	Hyperlipidemia	0.775	0.365	4.51	2.172 (1.062, 4.442)	0.034
Hematological indicators						
	NLRP1	0.013	0.004	9.173	1.013 (1.005, 1.022)	0.002
	ALP	0.013	0.006	3.883	1.013 (1.000, 1.026)	0.049
	TG	0.507	0.178	8.08	1.661 (1.171, 2.356)	0.004

Abbreviations: NLRP1, nucleotide-binding oligomerization domain like receptor protein 1; ALP, alkaline phosphatase; TG, triglyceride; CAC, coronary artery calcification.

### 3.3 Clinical Features of the Training Cohort and Validation Cohort

We divided the patients into a training cohort and a validation cohort in the ratio of 7:3 at random, to avoid overfitting of the model during analysis. Except for gender and total cholesterol (TC), no difference was found between the training set and the validation set, indicating comparability and rationality of division of our dataset (Table 3).

**Table 3.** **Baseline characteristics of training and validation sets**.

Variables		Training set (n = 436)	Validation set (n = 190)	*p-*Value
Age		63.26 ± 11.98	61.78 ± 10.45	0.140
Gender (n, %)				0.038
	Male 1	257 (58.9%)	95 (50.0%)	
	Female 2	179 (41.1%)	95 (50.0%)	
BMI, ${\mathrm{kg/m}}^{2}$				0.563
Smoking (n, %)				0.122
	No	287 (65.8%)	137 (72.1%)	
	Yes	149 (34.2%)	53 (27.9%)	
Drinking (n, %)				0.998
	No	319 (73.2%)	139 (73.2%)	
	Yes	117 (26.8%)	51 (26.8%)	
Past medical history (n, %)				
	Hypertension	243 (55.7%)	99 (52.1%)	0.402
	Diabetes mellitus	151 (34.6%)	58 (30.5%)	0.316
	Hyperlipidemia	135 (31.0%)	71 (37.4%)	0.117
Hematological indicators				
	NLRP1, pg/mL	48.09 (30.04, 68.34)	44.70 (28.03, 66.51)	0.295
	NLRP3, pg/mL	41.66 (19.50, 72.10)	44.04 (20.64, 68.76)	0.997
	WBC, ${\mathrm{10}}^{9}$/L	5.90 (4.90, 7.20)	5.60 (4.80, 7.10)	0.282
	NE, ${\mathrm{10}}^{9}$/L	3.55 (2.79, 4.55)	3.40 (2.79, 4.36)	0.408
	LY, ${\mathrm{10}}^{9}$/L	1.60 (1.30, 2.00)	1.60 (1.20, 2.00)	0.112
	PLT, ${\mathrm{10}}^{9}$/L	212.00 (179.00, 252.00)	207.50 (171.75, 237.25)	0.058
	hsCRP, mg/L	1.60 (0.50, 4.00)	1.70 (0.68, 5.40)	0.239
	AST, U/L	18.50 (15.00, 24.00)	18.00 (15.00, 24.25)	0.740
	ALT, U/L	17.00 (12.00, 27.00)	16.00 (12.00, 26.00)	0.443
	ALP, U/L	73.00 (60.00, 92.00)	70.00 (60.00, 89.00)	0.390
	Urea, mmol/L	5.18 (4.29, 6.38)	5.01 (4.17, 6.06)	0.182
	Scr, umo/L	62.00 (54.00, 72.00)	61.50 (53.00, 71.00)	0.434
	UA, umo/L	303.82 (247.05, 375.00)	289.00 (241.75, 352.25)	0.096
	eGFR, mL/min	105.12 (93.56, 117.76)	104.74 (89.25, 115.65)	0.264
	FBG, mmol/L	5.69 (5.21, 6.92)	5.68 (5.14, 6.77)	0.389
	TC, mmol/L	4.14 (3.52, 5.08)	3.93 (3.04, 5.00)	0.025
	TG, mmol/L	1.62 (1.17, 2.19)	1.55 (1.19, 2.10)	0.478
	LDL-C, mmol/L	2.59 (1.82, 3.30)	2.36 (1.83, 3.05)	0.063
	HDL-C, mmol/L	1.09 (0.93, 1.27)	1.05 (0.93, 1.27)	0.692
	APOA1, g/L	1.23 (1.09, 1.50)	1.24 (1.07, 1.44)	0.317
	APOB, g/L	0.89 (0.76, 1.08)	0.93 (0.80, 1.09)	0.204
	Lp (a), mg/L	232.00 (163.25, 339.75)	248.00 (148.75, 372.00)	0.871
	sdLDL, mmol/L	0.63 (0.45, 0.95)	0.70 (0.47, 0.98)	0.288
	NT-proBNP, pg/mL	160.39 (99.00, 440.43)	137.76 (99.00, 458.86)	0.457
	HbA1c, %	6.00 (5.60, 6.80)	5.90 (5.60, 6.50)	0.166
	LVEF, %	59.90 (56.00, 63.00)	59.44 (56.11, 63.23)	0.974
Inotropic drugs (n, %)				
	Statin	405 (92.9%)	173 (91.1%)	0.427
	Ezetimibe	78 (17.9%)	35 (18.4%)	0.874
	Apolizumab	62 (14.2%)	30 (15.8%)	0.610
	Aspirin	424 (97.2%)	183 (96.3%)	0.532
	Clopidogrel	142 (32.6%)	57 (30.0%)	0.526
	Ticagrelor	263 (60.3%)	111 (58.4%)	0.656
	ACEI/ARB	133 (30.5%)	55 (28.9%)	0.696
	ARNI	82 (18.8%)	37 (19.5%)	0.845
	Beta-blockers	263 (60.3%)	111 (58.4%)	0.656
	Calcium channel blockers	76 (17.4%)	35 (18.4%)	0.766
	Oral hypoglycemic drugs	138 (31.7%)	55 (28.9%)	0.501
	Insulin	25 (5.7%)	13 (6.8%)	0.593

Abbreviations: BMI, body mass index; NLRP1, nucleotide-binding oligomerization domain like receptor protein 1; NLRP3, nucleotide-binding oligomerization domain like receptor protein 3; WBC, white blood cell; NE, neutrophil; LY, lymphocyte; PLT, platelet; hs-CRP, high-sensitivity C-reactive protein; AST, aspartate aminotransferase; ALT, alanine transaminase; ALP, alkaline phosphatase; Scr, serum creatinine; UA, uric acid; eGFR, estimated glomerular filtration rate; FBG, fast blood glucose; TC, total cholesterol; TG, triglyceride; LDL-C, low-density lipoprotein cholesterol; HDL-C, high-density lipoprotein cholesterol; APOA1, apolipoprotein A; APOB, apolipoprotein B; Lp (a), lipoprotein (a); sdLDL, small dense low density lipoprotein; NT-proBNP, N-terminal-pro brain natriuretic peptide; HbA1c, glycated hemoglobin A; LVEF, left ventricular ejection fraction; ACEI, angiotensin-converting enzyme inhibitor; ARB, angiotensin receptor blockers; ARNI, angiotensin receptor neprilysin inhibitor; n, the number of patients per group.

### 3.4 Development and Validation of the Nomogram

**Fig. 3.**
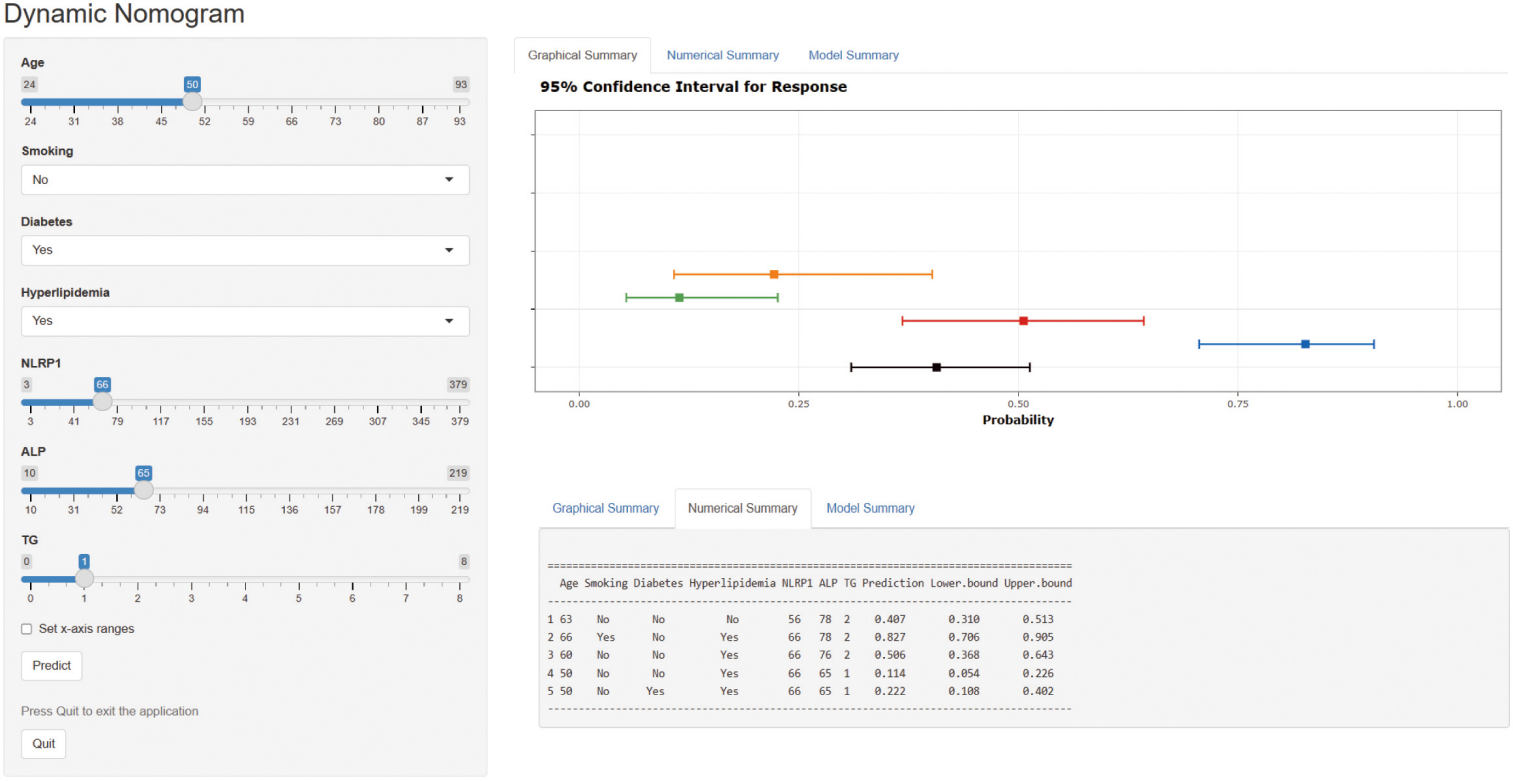
**Dynamic nomogram to predict the risk of CAC occurrence in patients with CAD.** Click on this link (https://jingfengpeng.shinyapps.io/DynNomapp/) to access the prediction model. Abbreviations: NLRP1, nucleotide-binding oligomerization domain (NOD)-like receptor protein 1; ALP, alkaline phosphatase; TG, triglyceride; CAC, coronary artery calcification; CAD, coronary artery disease.

**Fig. 4.**
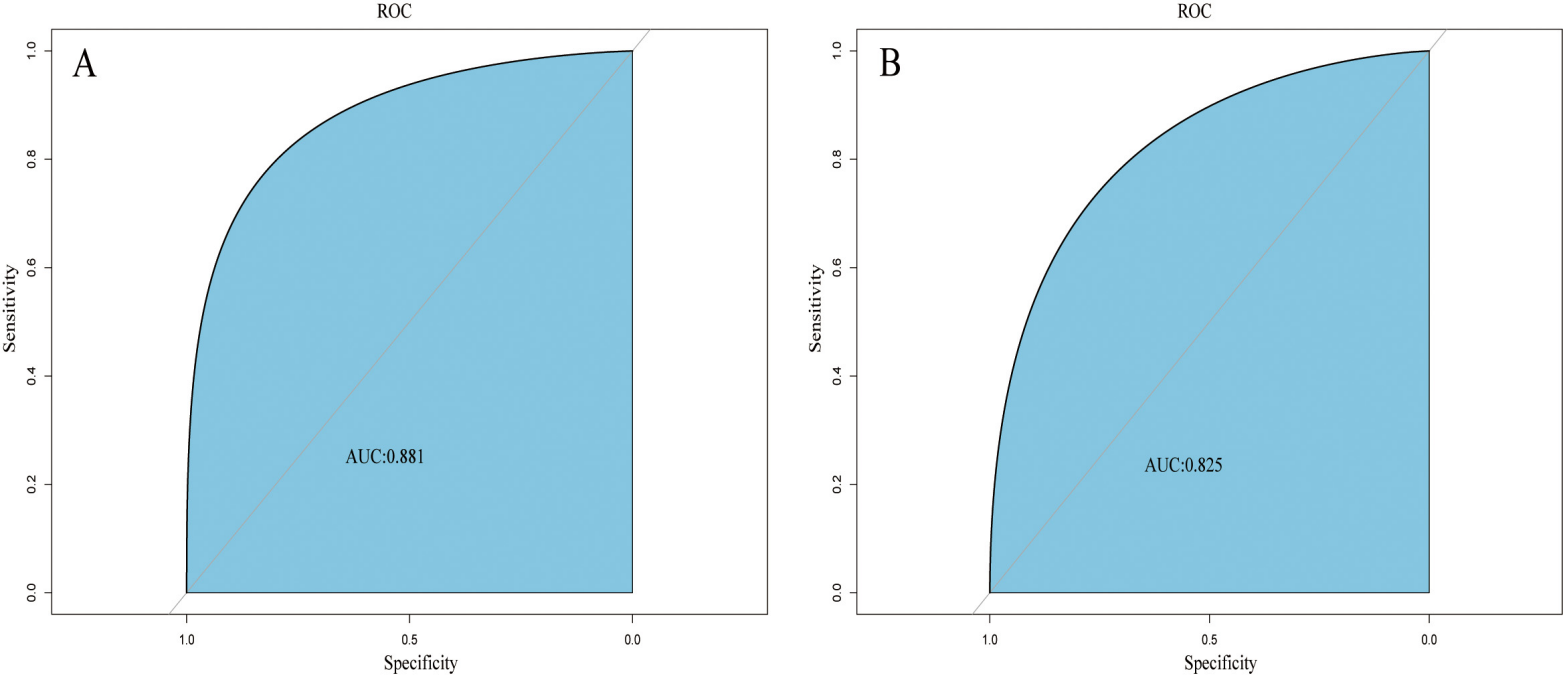
**ROC curves of the nomogram based on the training set (A) and validation set (B).** The AUC was utilized to judge the discriminative ability of model. Abbreviation: AUC, area under the ROC; ROC, receiver operating characteristic.

**Fig. 5.**
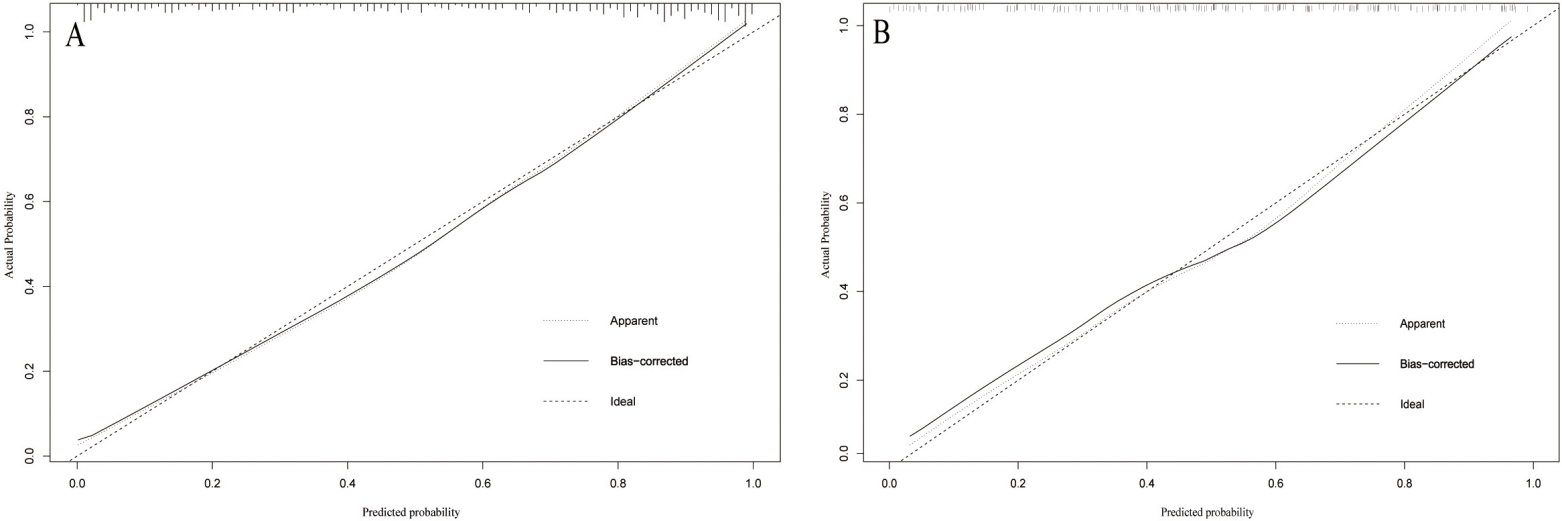
**Calibration curve of the model on the data of the training set (A) and validation set (B).** The diagonal 45-degree line indicates perfect prediction. Model calibration is represented by the degree of fitting of the curve and the diagonal line.

**Fig. 6.**
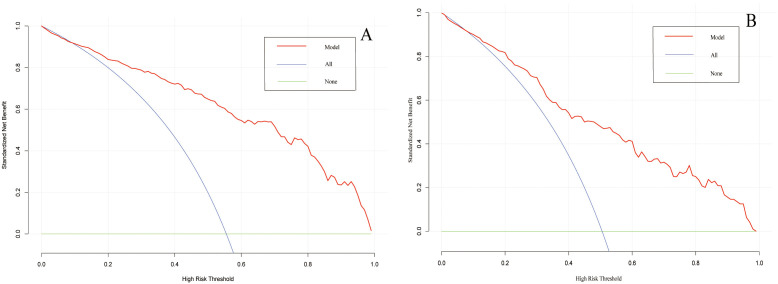
**Decision curve analysis of the prediction model on the data of the training set (A) and validation set (B).** The horizontal line and the oblique line respectively represent two extreme situations where all samples are negative, treated none and all samples are positive, treated all. The red curve represents the net benefit at each risk threshold.

A web-based dynamic nomogram (https://jingfengpeng.shinyapps.io/DynNomapp/) for predicting CAC occurrence was constructed by stepwise selection using the LASSO regression and multivariate logistic analysis (Fig. 3). We then applied the ROC curve to validate the model’s discriminative ability (Fig. 4). The AUC in both datasets were 0.881 (95% CI: 0.850–0.912) and 0.825 (95% CI: 0.760–0.876), respectively. This suggested that this nomogram had a favorable discriminative performance. In addition, we conducted the internal verification of the model using the Bootstrap method. The calibration curves indicated a favorable agreement between the predicted probability of this model and the actual probability, demonstrating a suitable calibration of the model (Fig. 5). As shown in Fig. 6, the DCA curves showed that the nomogram could achieve greater net benefit in both datasets than the two extreme cases, indicating the model has good clinical utility.

## 4. Discussion

Two key findings were identified in this study. First, the NLRP1 inflammasome was found to be an independent predictor of CAC. Second, we proposed and validated a valuable prediction model, embracing an extensive set of clinical risk factors that are easily accessible, such as age, smoking, DM, hyperlipidemia, ALP and TG, and incorporating the serum NLRP1 level. This web-based dynamic nomogram model (https://jingfengpeng.shinyapps.io/DynNomapp/) can soon be obtained for free online. The results generated from this model could serve as a guide for preventing or even slowing down the progression of CAC.

CAC plays an important role in CAD. The majority of mortality and major adverse events in cardiovascular disease are related to CAD, in which CAC plays a significant role. A significant correlation was found between the presence and extent of CAC and the overall magnitude of coronary atherosclerotic plaque burden, as well as the development of subsequent coronary events [3, 9]. Coronary calcification may deteriorate vascular compliance. Calcified plaques demonstrating a spotty pattern in coronary arteries are considered to increase the risk of plaque rupture [12, 13]. The progression of CAC, not only contributes to the risk of cardiovascular mortality, but also increases the difficulty for intervention therapy. CAC has always been a significant challenge for interventional cardiologists. Efforts to control CAC with medical therapy have not been successful. While advances in percutaneous techniques have modestly improved the outcomes of percutaneous coronary intervention (PCI), the risks and adverse events associated with the treatment of recalcitrant calcified lesions remains high. In addition, the treatment of CAC increases medical costs [6]. Therefore, it is necessary to expand therapies beyond mechanical revascularization to encompass predictive diagnosis and preventive interventions to treat CAC.

In patents with CAC, inflammation has been underestimated in previous prediction models. In recent years, emerging research suggest that the initiation and progression of CAC are collaboratively driven by long-term dyslipidemia and vascular inflammation, which are the basis of atherosclerosis [5, 9]. A study found that the greater extent of CAC among patients with severe rheumatoid arthritis was due to the effect of inflammatory mediators, which confirmed the strong impact of inflammation in the pathogenesis of CAC [14]. A major participant in the inflammatory response of CAC are macrophages, which further promote disease progression in a positive-feedback amplification loop of calcification and inflammation [9]. Proinflammatory stimuli induced by CVD promote the majority of inflammasome specks to accumulate in granulocytes and macrophages during the progression stage of inflammation [15, 16, 17]. We developed a strong interest in inflammasomes from earlier studies reports about inflammasomes and their derivation from macrophages [18, 19]. Several studies have found that there were numerous pattern-recognition receptors (PRRs) capable of assembling the inflammasome complex, but the well-established inflammasomes were still NLRP1, NLRP3, nucleotide-binding domain (NOD)-like receptor family caspase-associated recruitment domain-containing protein 4 (NLRC4) and absent in melanoma 2 (AIM2), among which NLRP1 and NLRP3 were the most widely studied in CVD [20]. Accumulating evidence supports that inflammasomes, capable of triggering and modulating inflammation-related signaling pathways, play the crucial role in the progression of various CVD [10, 21, 22, 23]. Studies have reported that the protein expressions of NLRP3 and caspase-1 in circulating monocytes among patients with acute coronary syndrome were increased [22]. Similarly, the NLRP1 inflammasome were found to increase in patients with primary atherosclerotic lesions and inflammasome complex was activated by interaction with NLRP1 and NLRC4 receptors [24]. In the progression of atherosclerotic lesions, cholesterol crystals were found to directly activate the NLRP3 inflammasome [25]. Elevated levels of triglycerides and very-low-density lipoprotein cholesterol stimulated activation of the NLRP1 inflammasome by nuclear factor kappa-B (NF-κB) [24, 26]. Additionally, interleukin-1β (IL-1β) and IL-18, downstream proinflammatory cytokines of inflammasomes, were also found to affect the development and stability of atherosclerotic plaques [27, 28]. These influencing factors subsequently combine lipid metabolism and inflammation to exacerbate disease progression. Therefore, we investigated whether NLRP1 or NLRP3 might be related to CAC. We found that a higher serum level of NLRP1 resulted in an increased CAC risk in patients with CAD. The NLRP3 inflammasome, the most widely explored inflammasome, was found to be unrelated to the prevalence of CAC. Further studies are needed to explain these novel findings.

Advanced age is a well-established risk and prognostic factor for CAC. Our logistic analysis revealed that patients with advanced age had a significantly increased risk of CAC, consistent with previous studies [3, 7]. Cigarette smoking, a significant health, remains highly prevalent worldwide and contributes to cardiovascular morbidity and mortality. Our findings, consistent with prior studies, showed that cigarette smoking was a critical factor of the presence and extent of CAC [29, 30]. Nicotine in cigarettes increases secretion of inflammatory cytokines and elevates lipid content within atherosclerotic lesions, subsequently causing osteogenic differentiation of vascular smooth muscle cells (VSMCs) [31]. CAC has also been found to be more severe in patients with DM [3, 8]. Our findings parallel prior studies on the relationship of DM and CAC. The mechanism of CAC induced by DM can be attributed to multiple factors. The main metabolites of diabetic individuals, advanced glycosylation end products (AGEs) can contribute to oxidative stress and the inflammatory response. Long-term exposure of VSMCs to a high glucose environment can activate relevant signaling pathways such as Wnt, extracellular signal-regulated kinases 1 and 2 (ERK1/2) and NF-κB, and increase the expression of Runt-related transcription factor 2 (Runx2) and Osterix (Osx), which are the key transcription factors that accentuates osteoblast‑like differentiation of VSMCs [5, 7, 32, 33]. In this study, we also found that a history of hyperlipidemia was significantly correlated with an increased risk for CAC. Experimental and clinical data have shown that hyperlipidemia not only promoted atherosclerotic plaque development, but also increased vascular calcification [4, 12]. The levels of oxidized phospholipids (ox-PLs) and oxidized low-density lipoprotein (ox-LDL) are elevated in the serum of patients with hyperlipidemia, which increases oxidative stress and the inflammatory response in the endothelium, as well enhancing the phenotypic transition of VSMCs into mature osteoblasts and mineralization by upregulating Osx expression, thereby leading to the initiation of CAC [34, 35]. Similar to our results, several animal experiments and clinical trials have confirmed a role of TG in the prevalence of CAC [35, 36]. Decreases TG levels can reduce the CAC Agatston score, a scoring method to estimate and quantify the extent of CAC [37, 38, 39]. This study also found that the serum ALP level was an independent risk factor of CAC. Several research studies have demonstrated that ALP, a key enzyme in vascular calcification, can hydrolyze phosphate bonds, inducing local accumulation of phosphate, which provides a microenvironment for calcification. Moreover, the activated ALP by various stimuli can provoke or modulate the osteoblast‑like differentiation of VSMCs [40, 41, 42].

## 5. Limitations

There are several limitations in this study. First, patients with certain diseases such as severe CKD, were more likely to prioritize receiving treatment from other departments, rather than undergoing CAG directly because of concern for increased complications. Lack of the data from these patients might affect the robustness of the model. Second, to prevent bias of insufficient data, some previously reported risk factors of CAC, such as the serum levels of parathyroid hormone (PTH), vitamin D, calcium and phosphorus, were not included. Third, this is a single-center study focused on the Chinese population. More information, such as ethnic background, diet and physical activity, awaits clarification in future studies to make the model more compatible and generalizable. In addition, to ensure the credibility and robustness of our model, more external data are warranted for validation in future studies.

## 6. Conclusions

This study found that the serum NLRP1 level was an independent risk factor of CAC in patients with CAD. We developed a web-based dynamic nomogram model consisting of 7 clinical characteristics, which may serve as a simple-to-use screening tool to personalize the risk of developing CAC and improve the therapeutic options for patients with CAD.

## Data Availability

All data that support the findings in this study are not publicly available due to patient privacy, but are available from the corresponding author upon reasonable request.
